# Supplementary material for: Seminoma and Embryonal Carcinoma Footprints Identified by Analysis of Integrated Genome-Wide Epigenetic and Expression Profiles of Germ Cell Cancer Cell Lines
Source: PLoS One. 2014 Jun 2;9(6):e98330. doi: 10.1371/journal.pone.0098330 (PMC4041891; doi:10.1371/journal.pone.0098330)
Supplement: File S1 — ZIP file containing DMRforPairs output for significant regions. Please start from the html files. (ZIP) [file pone.0098330.s008.zip › figures/28178.pdf]

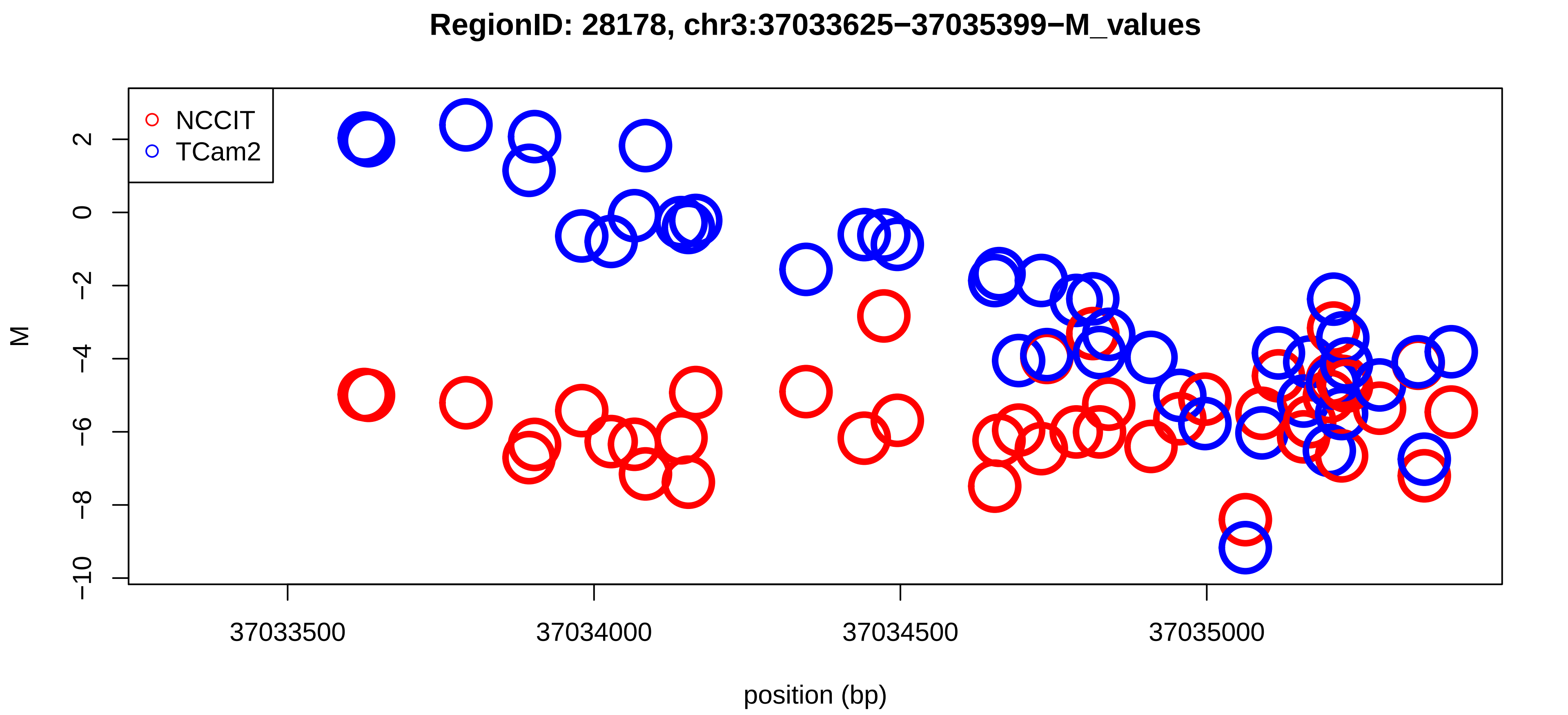

RegionID: 28178, chr3:37033625−37035399−M_values
NCCIT
TCam2
M
2
0
−2
−4
−6
−8
−10
37033500
37034000
37034500
37035000
position (bp)

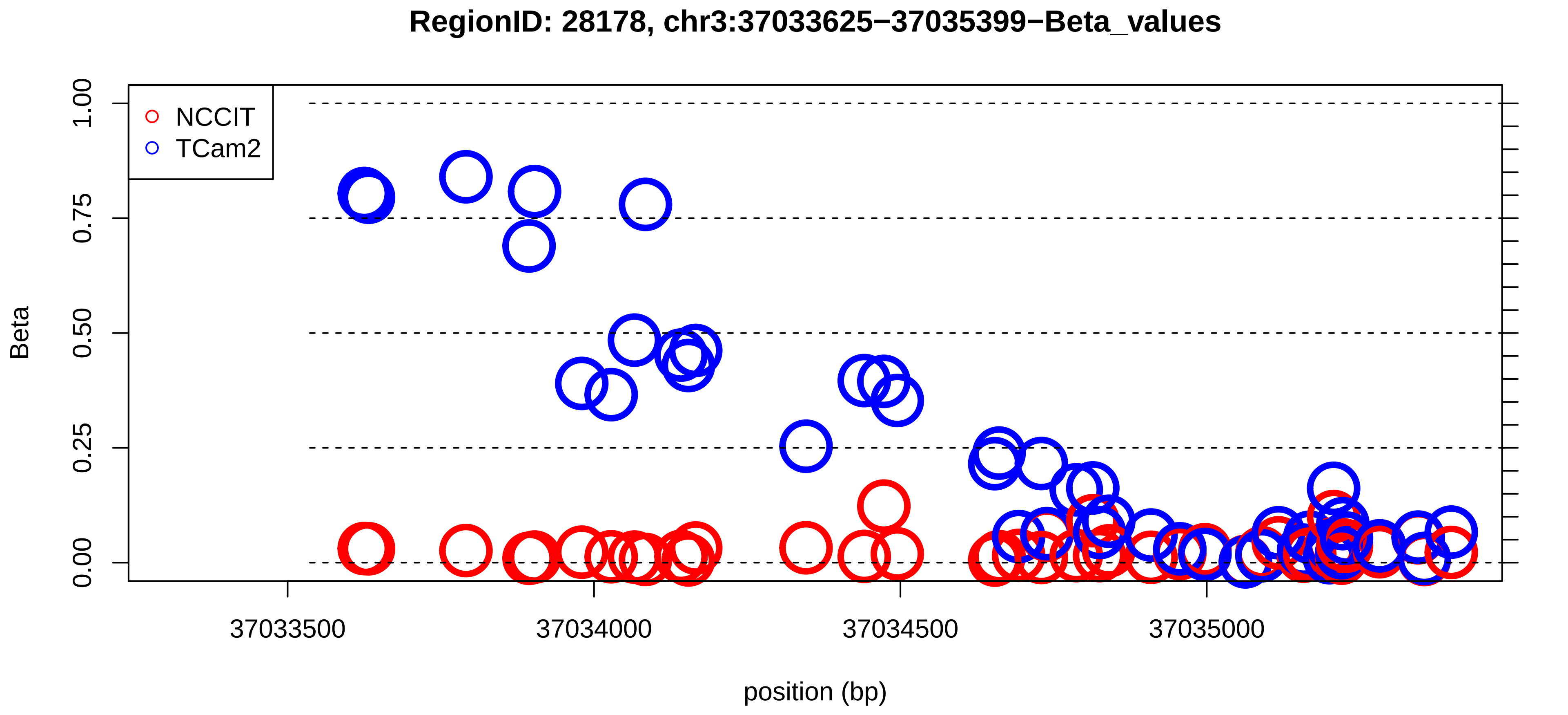

RegionID: 28178, chr3:37033625−37035399−Beta_values
NCCIT
TCam2
Beta
1.00
0.75
0.50
0.25
0.00
37033500
37034000
37034500
37035000
position (bp)

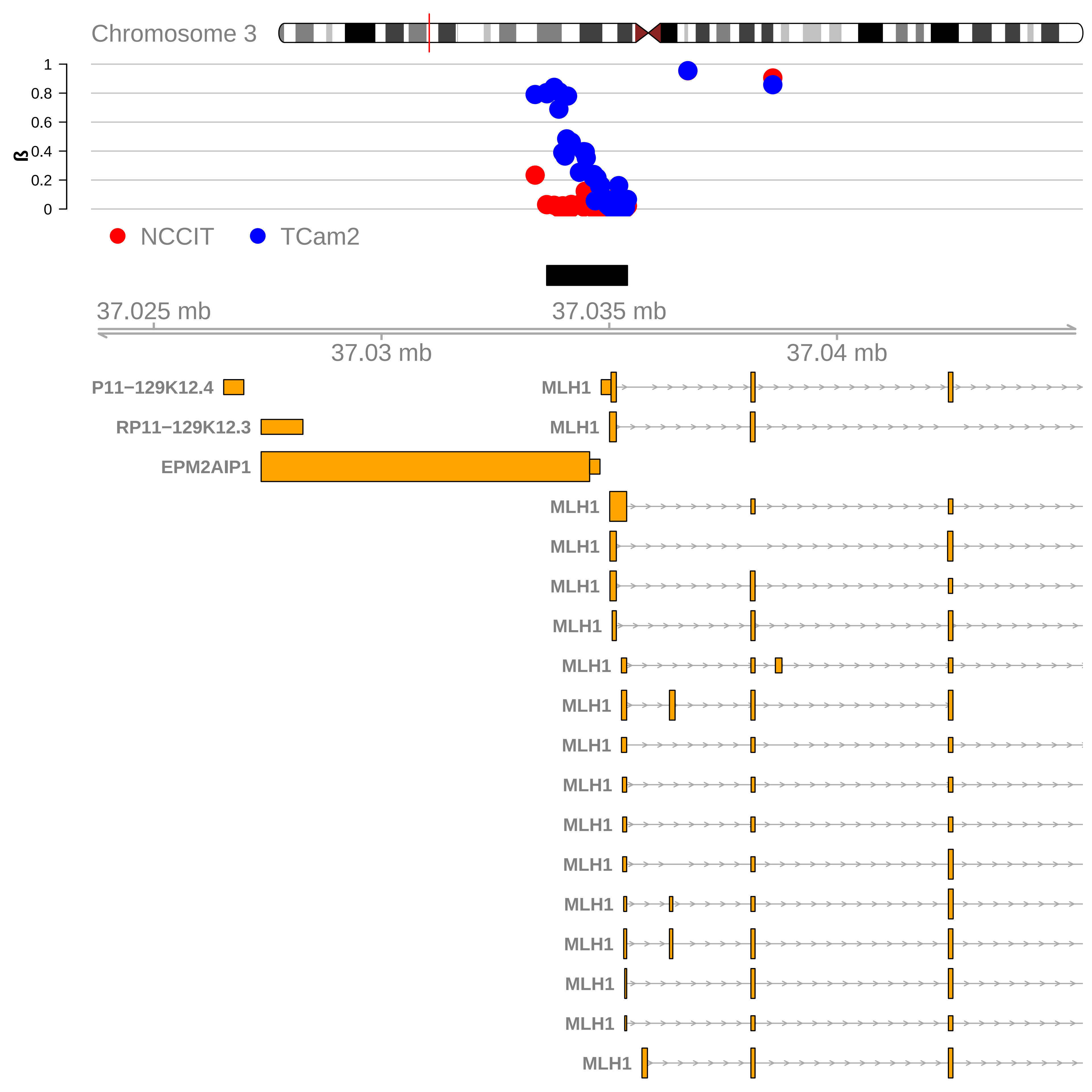

Chromosome 3
1
0.8
0.6
0.4
0.2
0
ß
NCCIT
TCam2
37.025 mb
37.035 mb
37.03 mb
37.04 mb
P11−129K12.4
RP11−129K12.3
EPM2AIP1
MLH1
